# Time perspective and well-being: Swedish survey questionnaires and data

**DOI:** 10.1016/j.dib.2016.08.057

**Published:** 2016-09-04

**Authors:** Danilo Garcia, Ali Al Nima, Erik Lindskär

**Affiliations:** a Blekinge Center of Competence, Blekinge County Council, Karlskrona, Sweden; b Department of Psychology, University of Gothenburg, Gothenburg, Sweden; c Institute of Neuroscience and Physiology, University of Gothenburg, Gothenburg, Sweden; d Network for Empowerment and Well-Being, Sweden; e Depeartment of Psychology, Lund University, Lund, Sweden

**Keywords:** Happiness, Negative affect, Positive affect, Psychological well-being, Temporal life satisfaction, Time perspective, Sweden

## Abstract

The data pertains 448 Swedes’ responses to questionnaires on time perspective (Zimbardo Time Perspective Inventory), temporal life satisfaction (Temporal Satisfaction with Life Scale), affect (Positive Affect and Negative Affect Schedule), and psychological well-being (Ryff׳s Scales of Psychological Well-Being—short version). The data was collected among university students and individuals at a training facility (see U. Sailer, P. Rosenberg, A.A. Nima, A. Gamble, T. Gärling, T. Archer, D. Garcia, 2014; [1]). Since there were no differences in any of the other background variables, but exercise frequency, all subsequent analyses were conducted on the 448 participants as one single sample. In this article we include the Swedish versions of the questionnaires used to operationalize the time perspective and well-being variables. The data is available, SPSS file, as Supplementary material in this article. We used the Expectation-Maximization Algorithm to input missing values. Little׳s Chi-Square test for Missing Completely at Random showed a *χ*^2^=67.25 (*df*=53, *p*=.09) for men and *χ*^2^=77.65 (*df*=72, *p*=.31) for women. These values suggested that the Expectation-Maximization Algorithm was suitable to use on this data for missing data imputation.

**Specifications Table**

**Table:** 

Subject area	*Psychology*
More specific subject area	*Time Perspective, Temporal Satisfaction with Life, Negative Affect, Positive Affect, and Psychological Well-Being, Happiness*
Type of data	*Swedish version of the questionnaires and SPSS file*
How data was acquired	*Paper and pencil and through online survey (distributed through Unipark)*
Data format	*Analyzed, missing values imputed*
Experimental factors	*The study had a cross-sectional design*
Experimental features	*The main variables were time perspective, temporal life satisfaction, positive affect, negative affect, and psychological well-being*
Data source location	*Sweden*
Data accessibility	*Data is within this article and as*Supplementary material

**Value of the data**

• This data can be employed for individual statistical analysis and meta-analysis.
• The data was collected in Sweden and has cultural diversity value.
• The questionnaires can be used for data collection among Swedish speaking participants.

## 1 Data

Questionnaires on time perspective (Zimbardo Time Perspective Inventory), temporal life satisfaction (Temporal Satisfaction with Life Scale), affect (Positive Affect and Negative Affect Schedule), and psychological well-being (Scales of Psychological Well-Being—short version) were answered by 453 individuals. Data screening and imputation left a total of 448 individuals [1].

## 2 Experimental design, materials and methods

### 2.1 Participants

The data were collected among 400 undergraduate students at a University in the West of Sweden (from which we obtained 324 valid responses corresponding to a 81% response rate) and 158 individuals at a training facility in the South of Sweden (from which we obtained 129 valid responses corresponding to a 82% response rate). The final sample consisted of 448 individuals (148 males and 300 females) with a mean age 29.74 years SD=12.86 years).

### 2.2 Questionnaires

*The Zimbardo Time Perspective Inventory* [2]. This instrument consists of 56 items (1=*strongly disagree*, 5=*strongly agree*) that measure five time dimensions: past positive (e.g., “It gives me pleasure to think about my past”), past negative (e.g., “I think about the good things that I have missed out on in my life”), present hedonistic (e.g., “Taking risks keeps my life from becoming boring”), present fatalistic (e.g., “Fate determines much in my life”), and future (e.g., “I believe that a person׳s day should be planned ahead each morning”). The Swedish version has been used and validated in previous and current studies [3], [4] and its psychometric properties validated in many different languages, such as Portuguese [5], Lithuanian [6], and Spanish [7]. See Appendix 1.

*The Temporal Satisfaction With Life Scale* [8]. This scale comprises 15-items rated on a 7-point Likert scale (1=*strongly disagree*, 7=*strongly agree*) organized in three subscales assessing past (e.g.,” If I had my past to live over, I would change nothing”), present (e.g.,”I would change nothing about my current life”), and future life satisfaction (e.g., “There will be nothing that I will want to change about my future”). The Swedish version has been validated in earlier and current studies [9]. See Appendix 2.

*The Positive Affect Negative Affect Schedule* [10]. This instrument requires participants to rate on 5-point adjective scales to what extent (1=*very slightly*, 5=*extremely*) during the last few weeks they experienced 10 positive and 10 negative affect. The positive affect scale includes adjectives such as strong, proud, and interested. The negative affect scale includes adjectives such as afraid, ashamed, and nervous. The Swedish version has been used in previous studies [11], [12], [13]. See Appendix 3.

*Ryff׳s Scales of Psychological Well-Being-short version* [14]. These scales consist of 18 items, 3 items for each of the six dimensions of psychological well-being: self-acceptance (e.g., “I like most aspects of my personality”), personal growth (e.g., “For me, life has been a continuous process of learning, changing, and growth”), purpose in life (“Some people wander aimlessly through life, but I am not one of them”), environmental mastery (e.g., “ I am quite good at managing the responsibilities of my daily life”), autonomy (e.g., “I have confidence in my own opinions, even if they are contrary to the general consensus”), and positive relations with others (e.g., “People would describe me as a giving person, willing to share my time with others”). The Swedish version has been used in previous studies [15], [16]. Since the subscales have been found to have low reliability, the total psychological well-being score (i.e., the sum of the 18 items) is recommended as a better and more reliable measure [15]. Appendix 4.

### 2.3 Statistical treatment

We first conducted an independent *t-test* between the undergraduate and the gym groups using the background variables (i.e., education, sleeping problems, exercise frequency, and use of psychotropic drugs) as the dependent variables. We assumed that non-significant differences between the samples in most of these variables would justify merging them into one single sample. The results showed that the groups only differed on exercise frequency (*t*=4.65, *df*=451, *p*<.001), the gym group reporting exercising more frequently (*M*=4.06) than the undergraduate group (*M*=3.59). Since there were no differences in any of the other background variables, all subsequent analyses were conducted on the 453 participants as one single sample.

We used the Expectation-Maximization Algorithm to input missing values. Little׳s Chi-Square test for Missing Completely at Random showed a *χ*^2^*=*67.25 (*df*=53, *p=*.09) for men and *χ*^2^=77.65 (*df*=72, *p*=.31) for women. This suggested that the Expectation-Maximization Algorithm was suitable to use on this data. There are significant correlations between many of the variables, which potentially introduces multicollinearity. Thus, the data were analyzed using Structural Equation Modeling (SEM) in order to control for error measurement and collinearity among variables. Based on our research questions we conducted two SEM models, one of the relationship between time perspective and temporal satisfaction and the other one of the relationship between time perspective and both affect and psychological well-being. *Kurtosis* and *skewness* values for all variables in the first (highest *kurtosis* value=.77; highest *skewness* value=−.65) and in the second (highest kurtosis value .77; highest skewness value .75) SEM model were within acceptable ranges.

## Figures and Tables

**Table:** 

	Stämmer inte alls	Stämmer inte särskilt bra	Stämmer delvis/delvis inte	Stämmer ganska bra	Stämmer precis

1. Jag anser att ett av livets stora nöjen är att festa med mina vänner.	1	2	3	4	5
2. Välbekanta syner, ljud och lukter från barndomen återuppväcker ofta en mängd underbara minnen.	1	2	3	4	5
3. Ödet bestämmer mycket i mitt liv.	1	2	3	4	5
4. Jag tänker ofta på vad jag borde ha gjort annorlunda i mitt liv.	1	2	3	4	5
5. Mina beslut påverkas av människor och saker omkring mig.	1	2	3	4	5
6. Jag anser att man bör planera sin dag i förväg varje morgon.	1	2	3	4	5
7. Att tänka på mitt förflutna ger mig glädje.	1	2	3	4	5
8. Jag gör saker impulsivt.	1	2	3	4	5
9. Om saker inte blir gjorda i tid, blir jag inte orolig.	1	2	3	4	5
10. När jag vill uppnå någonting sätter jag upp mål, och överväger specifika medel för att uppnå dessa mål.	1	2	3	4	5
11. Överlag har jag mycket mer bra än dåliga saker att minnas från mitt förflutna.	1	2	3	4	5
12. När jag lyssnar på min favoritmusik glömmer jag ofta bort tiden.	1	2	3	4	5
13. Möta morgondagens”deadlines” (sluttider) och att göra annat nödvändigt arbete går före kvällens nöjen.	1	2	3	4	5
14. Eftersom det blir som det blir, spelar det ingen roll vad jag gör.	1	2	3	4	5
15. Jag tycker om att lyssna på historier om hur det var på”den gamla goda tiden”.	1	2	3	4	5
16. Smärtsamma erfarenheter från det förflutna spelas upp i mitt huvud om och om igen.	1	2	3	4	5
17. Jag försöker leva mitt liv till fullo, en dag i taget.	1	2	3	4	5
18. Jag blir upprörd av att vara sen till möten.	1	2	3	4	5
19. Helst skulle jag vilja leva varje dag som om den vore min sista.	1	2	3	4	5
20. Lyckliga minnen från goda tider kommer lätt till mig.	1	2	3	4	5
21. Jag uppfyller mina förpliktelser till vänner och myndigheter i tid.	1	2	3	4	5
22. Jag har tagit emot min beskärda del av att känna mig illa bemött och avvisad i det förflutna.	1	2	3	4	5
23. Jag fattar beslut i stunden.	1	2	3	4	5
24. Jag tar dagen som den kommer hellre än att försöka planera den.	1	2	3	4	5
25. Det förflutna innehåller för mycket tråkiga minnen som jag helst inte vill tänka på.	1	2	3	4	5
26. Det är viktigt att skapa spänning i mitt liv.	1	2	3	4	5
27. Jag har gjort misstag i det förflutna som jag önskar att jag kunde ha ogjorda.	1	2	3	4	5
28. Jag anser att det är viktigare att njuta av det jag gör än att få jobb gjort i tid.	1	2	3	4	5
29. Jag blir nostalgisk över min barndom.	1	2	3	4	5
30. Innan jag fattar ett beslut väger jag in nackdelar mot fördelar.	1	2	3	4	5
31. Att ta risker gör att mitt liv inte blir tråkigt.	1	2	3	4	5
32. För mig är det viktigare att njuta av livets resa än att fokusera på vart jag är på väg.	1	2	3	4	5
33. Saker och ting blir sällan som jag har tänkt mig.	1	2	3	4	5
34. För mig är det svårt att glömma tråkiga minnen från min ungdom.	1	2	3	4	5
35. Att tänka på mål, utfall och produkter, tar udden av det jag gör.	1	2	3	4	5
36. Även när jag njuter av nuet, är jag benägen att jämföra med liknande tidigare erfarenheter.	1	2	3	4	5
37. I själva verket kan inte framtiden planeras eftersom saker och ting förändras så mycket.	1	2	3	4	5
38. Min livsväg är kontrollerad av makter jag inte kan påverka.	1	2	3	4	5
39. Det är meningslöst att oroa sig för framtiden, det finns ändå inget jag kan göra åt den.	1	2	3	4	5
40. Jag blir klar med arbetsuppgifter i tid genom att göra jämna framsteg.	1	2	3	4	5
41. Jag märker att jag slutar att lyssna när familjemedlemmar pratar om hur saker och ting brukade vara.	1	2	3	4	5
42. Jag tar risker för att tillföra mitt liv spänning.	1	2	3	4	5
43. Jag gör listor över saker att göra.	1	2	3	4	5
44. Jag lyssnar oftare på mitt hjärta än mitt huvud.	1	2	3	4	5
45. Jag kan motstå frestelser när jag vet att jag har arbete som måste bli gjort.	1	2	3	4	5
46. Jag dras med av stundens hetta.	1	2	3	4	5
47. Livet idag är för komplicerat, jag skulle föredra det enkla livet från förr.	1	2	3	4	5
48. Jag föredrar vänner som är spontana än de som är förutsägbara.	1	2	3	4	5
49. Jag tycker om familjeritualer och traditioner som upprepas regelbundet.	1	2	3	4	5
50. Jag tänker på de dåliga sakerna som har hänt mig i det förflutna.	1	2	3	4	5
51. Jag fortsätter att arbeta med svåra, ointressanta uppgifter om de hjälper mig att ligga steget före.	1	2	3	4	5
52. Att spendera det jag tjänar på njutningar idag är bättre än att spara för morgondagens trygghet.	1	2	3	4	5
53. Tur är oftast bättre än hårt arbete.	1	2	3	4	5
54. Jag tänker på de bra sakerna som jag har gått miste om i mitt liv.	1	2	3	4	5
55. Jag vill att mina nära relationer ska vara passionerade.	1	2	3	4	5
56. Det kommer alltid att finnas tid till att komma ikapp med mitt arbete.	1	2	3	4	5

**Table:** 

	**Inte alls**	**Lite**	**I någon mån**	**Ganska mycket**	**Väldigt mycket**
20. Engagerad	1	2	3	4	5
19. Förtvivlad	1	2	3	4	5
18. Upprörd	1	2	3	4	5
17. Haft skuldkänslor	1	2	3	4	5
16. Rädd	1	2	3	4	5
15. Fientlig	1	2	3	4	5
14. Entusiastisk	1	2	3	4	5
13. Stolt	1	2	3	4	5
12. Retlig	1	2	3	4	5
11. På alerten	1	2	3	4	5
10. Skamsen	1	2	3	4	5
09. Inspirerad	1	2	3	4	5
08. Nervös	1	2	3	4	5
07. Bestämd	1	2	3	4	5
06. Uppmärksam	1	2	3	4	5
05. Pirrig, ängslig	1	2	3	4	5
04. Aktiv	1	2	3	4	5
03. Skrämd	1	2	3	4	5
02. Ivrig	1	2	3	4	5
01. Stark	1	2	3	4	5

**Table:** 

		Stämmer Inte alls	Stämmer inte	Stämmer delvis inte	Stämmer delvis	Stämmer	Stämmer helt
18.	Att upprätthålla nära relationer har varit svårt och frustrerande för mig.	1	2	3	4	5	6
17.	Kraven i vardagslivet gör mig nedstämd.	1	2	3	4	5	6
16.	Jag känner mig på många sätt besviken med mina prestationer i livet.	1	2	3	4	5	6
15.	Jag tycker om de flesta delarna av min personlighet.	1	2	3	4	5	6
14.	Jag har inte upplevt många varma och tillitsfulla relationer med andra.	1	2	3	4	5	6
13.	Jag dömer mig själv utifrån vad jag tycker är viktigt, inte efter det andra anser vara viktigt.	1	2	3	4	5	6
12.	Generellt sett känner jag att jag har kontroll över min livssituation.	1	2	3	4	5	6
11.	Jag är bra på att hantera vardagens ansvar.	1	2	3	4	5	6
10.	Jag har en tendens att påverkas av människor med starka åsikter.	1	2	3	4	5	6
9.	Jag har slutat försöka göra stora förbättringar eller förändringar i mitt liv för länge sedan.	1	2	3	4	5	6
8.	Jag tycker att det är viktigt att få nya erfarenheter som utmanar vad jag anser om mig själv och omvärlden.	1	2	3	4	5	6
7.	Jag lever en dag i taget och tänker inte så mycket på framtiden.	1	2	3	4	5	6
6.	Jag har tilltro till mina egna åsikter, även om de skiljer sig från hur de flesta andra tänker.	1	2	3	4	5	6
5.	För mig har livet varit en ständig process av lärande, förändring och utveckling.	1	2	3	4	5	6
4.	Vissa människor går genom livet utan några mål, men jag är inte en av dem.	1	2	3	4	5	6
3.	Andra skulle beskriva mig som en givmild person, villig att ta sig tid för andra.	1	2	3	4	5	6
2.	Ibland känner jag att jag har gjort allt som finns att göra i livet.	1	2	3	4	5	6
1.	När jag ser tillbaka på mitt liv är jag nöjd med hur saker och ting har blivit.	1	2	3	4	5	6

## Appendix 1

### Zimbardo Time Perspective Inventory^1^

Läs varje påstående och svara så ärligt du kan på frågan:”Hur utmärkande eller sant är detta om dig?” Var vänlig och svara på samtliga frågor.

## Appendix 2

### Temporal Satisfaction With Life Scale^2^

Nedan finns 15 påståenden. Dessa påståenden har att göra med ditt förflutna, ditt liv just nu och din framtid. Använd den sjugradiga skalan nedanför för att ange ditt förhållningssätt till varje påstående genom att skriva lämplig siffra på raden framför. Var vänlig och svara så öppet och ärligt du kan.

7 – Håller verkligen med
6 – Håller med
5 – Håller med till viss del
4 – Håller varken med eller inte med
3 – Håller till viss del inte med
2 – Håller inte med
1 – Håller verkligen inte med

_____ 1. Om jag kunde leva om mitt förflutna skulle jag inte ändra på något.
_____ 2. Jag är nöjd med mitt förflutna.
_____ 3. Mitt förflutna har varit perfekt för mig.
_____ 4. Omständigheterna i mitt förflutna har varit utmärkta.
_____ 5. I mitt förflutna har jag haft allt som varit viktigt för mig.
_____ 6. Jag vill inte ändra på något i mitt liv just nu.
_____ 7. Jag är nöjd med mitt liv just nu.
_____ 8. Mitt liv just nu är perfekt för mig.
_____ 9. De nuvarande omständigheterna i mitt liv är utmärkta.
_____ 10. Jag har allt som är viktigt för mig just nu.
_____ 11. Det kommer inte att finnas något i min framtid som jag kommer att vilja ändra på.
_____ 12. Jag kommer att vara nöjd med mitt liv i framtiden.
_____ 13. Jag tror att min framtid kommer att vara perfekt för mig.
_____ 14. Omständigheterna i min framtid kommer att vara utmärkta.
_____ 15. I framtiden kommer jag att ha allt som är viktigt för mig.

## Appendix 3

### Positive Affect Negative Affect Schedule^3^

Den här skalan består av ett antal ord som beskriver känslor och stämningslägen. Ange i vilken utsträckning du har känt dessa under de senaste veckorna. Det gör du genom att läsa varje ord och ringa in det svarsalternativ som du tycker passar bäst in på dig.

## Appendix 4

### Ryff׳s Psychological Well-Being Scales^4^

Detta är en enkät som har att göra med hur du tänker i olika situationer. Läs igenom nedanstående påståenden och avgör hur mycket du håller med vart och ett enligt dina värderingar och erfarenheter.
