# Supplementary material for: Time perspective and well-being: Swedish survey questionnaires and data
Source: Data Brief. 2016 Sep 4;9:183–93. doi: 10.1016/j.dib.2016.08.057 (PMC5021799; doi:10.1016/j.dib.2016.08.057)
Supplement: Supplementary material [file mmc2.docx]

**Declaration of interest**

Dr. Danilo Garcia is the Director of the Blekinge Center of Competence, which is the Blekinge County Council’s research and development unit. The Center works on innovations in public health and practice through interdisciplinary scientific research, person-centered methods, community projects, and the dissemination of knowledge in order to increase the quality of life of the habitants of the county of Blekinge, Sweden. He is also an Associate Professor at the University of Gothenburg and together with Professor Trevor Archer and Associate Professor Max Rapp Ricciardi, the leading researcher of the Network for Empowerment and Well-Being. Ali Al Nima is a statistician and Erik Lindskär is a research assistant at the Blekinge Center of Competence. Both are also members of the Network for Empowerment and Well-Being.
